# Chimeric Antigen Receptor T Cell Therapy in Acute Myeloid Leukemia: Trials and Tribulations

**DOI:** 10.3390/hematolrep15040063

**Published:** 2023-11-12

**Authors:** Swati Garg, Wei Ni, James D. Griffin, Martin Sattler

**Affiliations:** 1 Department of Medical Oncology, Dana-Farber Cancer Institute, Boston, MA 02215, USA; wei_ni@dfci.harvard.edu (W.N.); james_griffin@dfci.harvard.edu (J.D.G.); martin_sattler@dfci.harvard.edu (M.S.); 2 Department of Medicine, Harvard Medical School, Boston, MA 02115, USA

**Keywords:** acute myeloid leukemia (AML), chimeric antigen receptor (CAR), adoptive cell therapy (ACT), CAR T, CAR NK

## Abstract

Acute myeloid leukemia (AML) is a heterogeneous hematological malignancy that is often associated with relapse and drug resistance after standard chemotherapy or targeted therapy, particularly in older patients. Hematopoietic stem cell transplants are looked upon as the ultimate salvage option with curative intent. Adoptive cell therapy using chimeric antigen receptors (CAR) has shown promise in B cell malignancies and is now being investigated in AML. Initial clinical trials have been disappointing in AML, and we review current strategies to improve efficacy for CAR approaches. The extensive number of clinical trials targeting different antigens likely reflects the genetic heterogeneity of AML. The limited number of patients reported in multiple early clinical studies makes it difficult to draw conclusions about CAR safety, but it does suggest that the efficacy of this approach in AML lags behind the success observed in B cell malignancies. There is a clear need not only to improve CAR design but also to identify targets in AML that show limited expression in normal myeloid lineage cells.

## 1. Introduction

Immune cells work in concert to fight off foreign pathogens, eliminate aberrant cells, or contribute to wound healing. During malignant transformation, this harmony between immune cells can be disrupted, and cancer cells escape detection by the body’s immune system. While innate immune cells are mainly involved in warding off microbial infections and parasites, they may also target some tumor cells [1]. The adaptive immune system is involved in the recognition of processed intracellular antigens, antigen clearance, memory response, and immune regulation through B cells and T cells. The association between the immune state of a person and the progression of a malignant manifestation has been known for a long time. The concept of immune therapy in cancer was first documented in the late 19th century, when tumor regression was observed in some sarcoma patients after erysipelas infection [2]. The importance of an active immune system in cancer surveillance became prominent in the 1960s after the discovery of the major effectors of the immune system. Conversely, numerous reports were published of an increased incidence of cancer in patients with immune deficiencies [3,4]. The effect of adoptive cell transfer (ACT) on tumor burden was first observed in patients who received allogeneic hematopoietic stem cell transplant (HSCT) for the treatment of hematological malignancies and subsequently showed immune reconstitution along with reduced leukemia burden. Since then, several different types of adoptive cell transfer of immune cells have been developed as treatment strategies against different types of malignancies [5]. 

In acute myeloid leukemia (AML), myeloid lineage blood-forming cells are arrested at different developmental stages (often referred to as ‘blasts’) and grow uncontrollably, which results in rapid hematopoietic insufficiency and bone marrow failure. AML is a heterogeneous cancer that generally affects older adults, with a median age of diagnosis of 65 years. Due to its diverse biology, wide spectrum of chromosomal aberrations, and mutational landscape, the clinical outcomes after standard induction treatment with daunorubicin and cytarabine are somewhat dismal, especially for patients older than 65 years of age, where the 5-year overall survival for this population is still below 10–15% [6]. For a subset of AML patients, HSCT after frontline induction chemotherapy has proven to be the most effective treatment, and tumor-reactive T cells from the donor are believed to be a major co contributors to this success [7]. 

There has been an intense effort to develop drugs that directly target the oncogenes that cause AML, and some successes have been noted. Acute promyelocytic leukemia (APL) with a PML-RARα fusion oncogene is so far the only AML subgroup where such targeted agents can be used successfully without concomitant chemotherapy. In APL, all-trans-retinoic acid (ATRA) and arsenic trioxide (ATO), two drugs that inhibit PML-RARα, can be used without standard chemotherapy for disease management with a high rate of complete remission and cure. Other new targeted agents have been shown to have survival benefits in selected AML subsets when added to standard chemotherapy regimens, including inhibitors of mutant FLT3 (midostaurin, sorafinib, gilteritinib, quizartinib, and others), IDH1 (ivosidenib), and IDH2 (enasidenib). These and other reagents in clinical development are often combined with the BCL2-targeting venetoclax to improve the induction of cell death in AML.

The development of immunotherapies for AML has also attracted considerable interest. For example, several monoclonal antibodies and immunotoxins have been tested in clinical trials, although so far, only anti-CD33 immunotoxins have had enough success to be approved by the FDA. In contrast, limited studies with checkpoint inhibitors to target CTLA4, PD-1, or PDL1 have not demonstrated significant activity by themselves in AML, although the number of trials is modest. Even with the advancements in our understanding of the disease and current guidelines for the diagnosis and management, survival remains poor, in particular in older patients [8,9], seer.cancer.gov (accessed on 1 June 2023). Given the successes observed with CAR T cell therapy in B cell tumors, including multiple types of lymphomas and acute lymphoblastic leukemias, there has been great interest in testing various CAR T cell products in AML, and in this review, we will discuss the current state of CAR T cell therapy in this disease. 

## 2. How Do Immune Cells Work?

The immune system comprises myeloid (monocytes, macrophages, and granulocytes), lymphoid (B, T, and natural killer cells (NK)), and dendritic cells (DC). As the first response, common molecular patterns on the surface of pathogenic bacteria or cells are recognized by the cells of the innate immune system, such as monocytes, DCs, granulocytes, NK, and innate lymphoid cells, which lead to the activation of immune components, including adaptive immune cells such as B and T cells. While monocytes, macrophages, granulocytes, and B cells are generally involved in phagocytosis of pathogens and antigen presentation, cytotoxic T cells and NK cells have direct cytolytic functions. The three major lymphoid cells have specific functions: B cells are specialized APCs that secrete antigen-neutralizing antibodies and are involved in immune activation. NK cells can detect a variety of stress-induced self and infectious non-self ligands to activate cytotoxicity through the release of lytic granules in target cells and the release of cytokines for immune activation. T cells have immune-activating, regulatory, and direct cytolytic roles in immune reactions [10,11,12,13]. CAR therapy aims to harness the direct cytolytic function of cells, and therefore T and NK cells are the major cell types used for CAR engineering.

**Figure 1:**
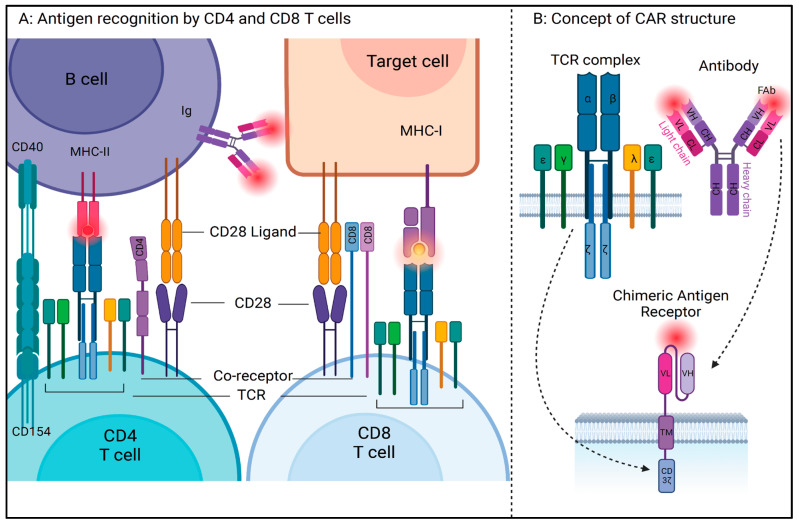
(**A**) Antigen recognition by CD4 and CD8 T cells in conjunction with MHC complex expressed on antigen-presenting cells. (**B**) Concept of CAR structure: To circumvent the MHC restriction of antigen recognition by T cells, synthetic receptor containing the complementarity-determining regions (CDR) on variable light and heavy (vH and vL) chains of monoclonal antibodies are cloned into a single variable fragment (scFv), which is then attached to the transmembrane and intracellular signaling domains of T cell receptor components via hinge region (created with BioRender (https://www.biorender.com/, accessed on 17 July 2023)).

T cells play a central role in the immune system by establishing successful cell-based immune responses. They recognize antigen fragments presented by the major histocompatibility complexes (MHC) on the surface of antigen-presenting cells (APCs) through the T cell receptor (TCR) complex. Most T cells in the periphery express TCR made of alpha/beta glycoproteins, and a minority of T cells express gamma/delta TCR. These glycoproteins belong to the immunoglobulin (Ig) superfamily, and like the Igs, TCRs have constant and variable regions, transmembrane and cytoplasmic domains, and the genes encoding TCRs undergo similar splicing events as those of B cell membrane immunoglobulins. However, unlike Igs, TCRs can only identify an antigen when presented with MHC. Of note, in CAR T cells, the MHC restriction is removed by substituting the TCR recognition function with an Ig-like element. The TCR complex itself consists of non-covalently linked CD3 chains that form heterodimers comprising epsilon-gamma (εγ), epsilon-delta (εδ), and zeta-zeta (ζζ) signaling chains that form an octamer TCR complex [14,15]. A mature T cell in the periphery expresses a clone-specific TCR complex along with one of the two co-receptors, CD4 or CD8, that determine its MHC restriction [16,17]. CD4 T cells recognize mainly exogenous, or pathogen-derived antigens such as bacterial peptides presented in the MHC II complex on antigen-presenting cells. T helper or CD4 T cells orchestrate immuno-protective and regulatory functions by releasing inflammatory cytokines, chemokines, trans phagocytosis, and recruitment of other immune cells [18,19]. CD8 T cells mainly recognize self-derived antigens presented with MHC class I complexes on the antigen-presenting cells [20,21]. Since MHC I is expressed on almost all nucleated cells, virally infected and tumor-associated antigens are efficiently captured via CD8 T cells, which have direct cytolytic function upon activation. There are additional T cell types defined by their functionality, differential antigens, and cytokines. Figure 1A depicts the general concept of antigen recognition by CD4 and CD8 T cells. While TCR and co-receptor engagement are sufficient for the initial priming, antigen recognition, and thymic selection, a costimulatory signal through CD28 is required for optimal T cell activation. The B7 family of immunomodulatory receptors, including CD28 ligands B-7, CD80 (B7-1) and CD86 (B7-2) are expressed on APCs and play an important role at immunological synapses (IS). CD28 mediates the full activation of T cells by organizing the membrane rafts to IS through activation of a series of signaling events that include phosphorylation of key immunoreceptor tyrosine-based activation motifs (ITAM) [22]. Subsequently, T cell activation through CD28 engagement induces the expression of cytotoxic T lymphocyte-associated antigen-4 (CTLA-4; CD152) on its surface. CTLA-4 has a higher affinity for B7-1 and B7-2 than CD28, and it is responsible for trans-endocytosis and downregulation of CD28 ligands, disturbance of IS via cytoskeleton restructuring, and recruitment of several phosphatases to dampen the activated state of T cells. CTLA-4 thus exerts a co-inhibitory function and creates a suppressive and regulatory state. In a typical immune response, T cell-antigen priming and activation are followed by regulation, anergy, and exhaustion to create clearance, memory, self-tolerance, and prevention from auto- or hyper-immune reactions. These are important regulatory functions required to retain normal T cell homeostasis, and current CAR strategies attempt to optimize these functions. Additional co-stimulatory and co-inhibitory molecules have been identified to play a role in T cell function and are reviewed in greater detail in the literature [23,24,25].

## 3. How Do Cancer Cells Evade the Immune System?

According to the cancer immunosurveillance hypothesis and its addendums, immune cells can recognize and regulate the growth of transformed malignant cells, but they can also allow malignant cells with low immunogenicity to outgrow. Cancer cells are known for immunoediting through several mechanisms at multiple levels. As discussed earlier, for a T cell to recognize a self-transformed cell, an antigen has to be presented through the MHC-I complex; downregulation of MHC-I and other components of antigen presentation pathways serve as an important immune escape mechanism for several tumors [26]. Cancer cells can also escape immune recognition by lowering their antigenicity, whereby unless the transformed cell expresses a neo or aberrant antigen at high levels, it cannot be recognized by the cells of the immune system. Cancer cells can achieve a reduction in immunogenicity by dampening T cell activation and inducing regulatory function via overexpression of immune checkpoint ligands such as PD-L1, MHC-II, FGL1, Galectin-9, and HMGB1 to engage PD1, LAG3, and TIM3 on T cells [27,28,29]. The cytokine composition of the tumor microenvironment can play a repressive role for immune helper cells such as dendritic cells (DCs) by releasing IL10, prostaglandin-2, TGF-beta, and indoleamine 2,3-dioxygenase (IDO) [30]. Type I interferon-induced epigenetic reprogramming for increased stemness and immune escape in cancer cells is yet another mechanism with which cancer cells can render normal immune cells non-functional against them [31,32]. This is not a comprehensive list of all immune escape mechanisms, but in general, immunotherapy and CAR cell therapy attempt to circumnavigate many of these obstacles to arrive at an activated anti-tumor-specific state. 

## 4. What Is a Chimeric Antigen Receptor (CAR) T Cell? 

CAR T cells are genetically engineered T cells that express an exogenous, synthetic receptor able to recognize a specific antigen in the absence of MHC restriction, leading to optimized T cell activation for effective target cell killing. The concept of CAR T cells first came to light in 1987 when Dr. Zelig Eshhar retrovirally infected T cells to express an engineered TCR with the goal of killing tumor cells. In the late 1980s, several groups, including Dr. Steven Rosenberg at the NCI, Dr. Carl June at Penn Medicine, Dr. Michel Sadelain at MSKCC, Dr. Dario Campana at St. Jude, and others, tried to develop more effective CAR T cells. By the end of the 1990s, the groups of Dr. Sadelain and Dr. June showed the translational optimization of engineered T cells for clinical application, crediting Dr. June as the pioneer of CAR T therapy [33,34]. 

## 5. What Is the Structure of a CAR?

Conceptually, prototype CAR T receptors require four main structural components in order to sustain an effective T-cell-mediated immune response [35] (Figure 1B), including the following:

1. Antigen-Binding Domain: The discovery of monoclonal antibodies in the 1970s played an important role in the conceptualization of antigen specificity using complementary determining regions of variable and constant regions of immunoglobulins [36]. In a CAR antigen, the antigen-binding domain is a single-chain variable fragment (scFv), derived from variable light (vL) and heavy chains (vH), and a flexible linker of the antigen-specific monoclonal antibody. The usage of antibody-mediated antigen recognition systems allows the CAR T cell to circumvent MHC restriction;
2. Hinge Region: This region connects the antigen-binding domain to the transmembrane domain and affects the overall steric conformation of the CAR to the antigen. These can be of various lengths and are generally derived from the sequence of T cell coreceptors such as CD8, CD28, or immunoglobulins. Shorter extracellular domains increase the potential for CAR T activation, whereas lengthening the CAR antigen diminishes CAR T activation [37];
3. Transmembrane Domain: This domain is the region that anchors the CAR to the T cell membrane. Generally, TM domains are derived from amino acid sequences of T cell coreceptors such as CD4, CD8, CD3zeta, and CD28 and are reported to be involved in cytokine release and cell death, apart from their overall stability. The stability of CAR and its expression on the T cell membrane is affected by the transmembrane domain, whereas the hinge domain is critical in the regulation of signaling threshold [38];
4. Intracellular Signaling Domain: This domain, also called the costimulatory (CM) domain, transduces the signaling cascade and is involved in T cell activation after successful antigen recognition. This is the domain that contains the necessary ITAMs for downstream signaling cascade activation. Because of its role in cell stimulation, cytokine release, and activation-induced cell death, the intracellular domain has been the most focused of all the regions over the years through multiple generations of CAR antigens.

The DNA sequences encoding these domains of a given CAR are then used to genetically engineer autologous or allogeneic T cells [39,40,41]. Transgene delivery to T cells is carried out using viral vectors such as adenovirus, lentivirus, or retrovirus; alternatively, electroporation-, nuclease-, polymer-, or lipid-nanoparticle-based gene delivery systems are used [42]. 

## 6. How Did the CAR Design Evolve?

Over the years, CAR designs have emerged to improve efficacy, reduce toxicity, increase safety, and overcome the limitations posed by the tumor microenvironment, tumor immune evasion, and relapse. Various CAR designs have shown their promise in experimental and clinical settings. The basic structural concept of the CAR molecule remains the same, but different domains and sequences have shown differential activity and efficacy. The first-generation CAR incorporated a single intracellular domain derived from the zeta chain of CD3 and lacked persistent in vivo proliferation. To overcome the challenge of limited in vivo proliferation, in the second-generation CAR, one additional costimulatory domain was added to the intracellular region, which was derived from either CD28 or 4-1BB. These CARs showed better proliferation in the absence of exogenous signals. To achieve optimal activation, additional ITAMs were designed in the third-generation CAR, where two additional intracellular domains from the receptors, such as CD28, 4-1BB, CD27, CD40, OXO-4, DAP-12, or ICOS, were used [43,44]. An addition of these intracellular receptor domains further enhanced the stimulation and provided better regulation of CAR T survival, proliferation, and tumor killing efficiency. While third-generation CAR is considered the optimal CAR design for early clinical trials, next-generation CAR strategies are built upon second-generation CAR. 

## 7. What Are the Challenges and Strategies to Overcome Shortcomings in CAR Design?

**Figure 2:**
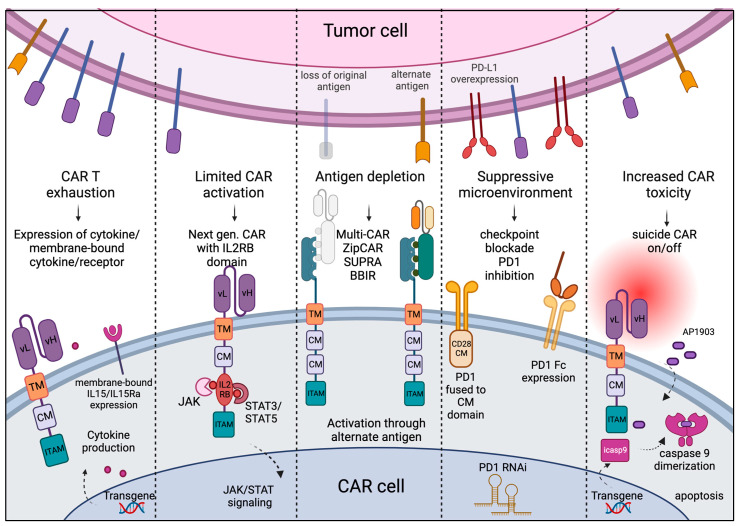
Challenges and approaches: Cell exhaustion, limited activation, and increased off-target toxicity of CAR cells can be regulated by providing cytokine transgenes in armored CARs, adding IL2-receptor-binding domain to enhance JAK/STAT signaling for T cell activation, and off-signaling in response to dimerizing agents to dampen the toxicity. Use of universal CAR design can circumvent the problem of antigen depletion and outgrowth of tumor population with alternate antigens, and use of immune checkpoint blockade can overcome the suppressive tumor microenvironment. (Created with BioRender.)

Despite the seemingly straight-forward immune reaction required to kill tumor cells using engineered CAR T cells, challenges arise whereby CAR T cells cannot recognize escaped tumor cells that relapse in either the same or altered phenotype, or CAR treatment results in acute toxicity leading to life-threatening conditions. Therefore, numerous strategies are utilized to optimize CAR design to address specific challenges (Figure 2).

- **CAR T exhaustion:** Patients receiving CAR treatment may have suboptimal immune composition due to overt tumor burden or previous therapies. CAR T cells can be rapidly exhausted in vivo with limited trans-presented cytokines and a lack of helper signaling from other immune cells such as APCs and NK cells. To overcome this challenge, CAR T cells are engineered with transgenes to produce functional cytokines under the control of the Nuclear Factor for Activated T Cells (NFAT) promoter, which enables CAR T cells to recruit other immune cells. These CARs are also known as TRUCKs, or T cells Redirected for Universal Cytokine-mediated Killing, and express the cytokine transgene encoding either IL-12, IL-18, TNFRSF14 [45], or membrane-bound IL-15 [46]. Other strategies, such as pre-treatment of T cells with IL-7, IL-15, or IL-21 in culture prior to adoptive cell transfer (ACT) or in vivo inhibition of the PI-3/AKT pathway using small molecule inhibitors, are shown to prevent T cell exhaustion [47].
- **CAR T-mediated toxicity and fratricide:** In most cases, the antigen against which the CAR is developed is not truly a tumor-exclusive antigen and is expressed by the cells of normal tissues as well. This causes T cell-mediated on-target off-tumor toxicity. When the antigen of interest is also expressed with an activated CAR cell, there is a possibility of one CAR cell killing another, resulting in fratricide. A generalized, non-specific immune activation and acute toxicity have also been observed in several CAR T clinical trials. In fourth-generation CAR T cells, transgene coding for proteins that lead to CAR T apoptosis or shut-down in response to a specific ligand is incorporated to prevent CAR-mediated toxicity. Two frequently used suicide or off switches are herpes simplex virus thymidine kinase (HSV-TK), inducible with ganciclovir, and inducible caspase-9 (iCasp9) that dimerizes after the administration of AP1903 [48]. CRISPR-mediated deletion of the CAR target gene from the CAR cells is generally used to overcome the issue of fratricide in CAR cells.
- **Suboptimal CAR activation and terminal differentiation:** Optimal CAR activation is required for anti-tumor activity and longer persistence of CAR T cells in vivo. In the fifth or next-generation CAR, the intracellular CD3 zeta and CD28 costimulatory signal is accompanied by a truncated IL2 receptor beta chain cytoplasmic tail with STAT3 binding sites, which can recruit docking of transcription factors and activation of JAK/STAT signaling in response to antigen binding. This modification enhanced CAR T persistence and proliferation and prevented their terminal differentiation [49].
- **Antigen escape:** A common challenge in cancer is that tumor cells can shed or downregulate the expression of antigens, and after the initial clearance of the major tumor population, resistant cells without the target antigen or with an alternate antigen can outgrow. This posed a challenge in the conventional CAR design as they could only recognize one single antigen at a time. One way to overcome this challenge is a multiplexed or universal CAR strategy where the conventional single-chain variable fragment (scFv) is replaced with an adapter-specific recognition domain that binds to an adaptor that is ligated to tumor-specific antigens [50]. A split, universal, and programmable (SUPRA) is a two-component receptor system composed of a universal receptor (zipCAR) expressed on T cells and a tumor-targeting scFv adaptor (zipFv), which, when binds to tumor-specific antigens, can ligate to ZipCAR and mediate efficient tumor killing [51]. Another such strategy is to use biotin-binding immune receptor (BBIR) [52] or Bi-specific T engagers, or CART.BiTE, to target heterogenous antigen-expressing tumors [53].
- **Suppressive tumor microenvironment:** Tumor cells can express several inhibitory signals, such as PD-L1, that may lead to inhibitory signaling through PD1 on the engineered T cells, resulting in their rapid exhaustion. Several strategies are being used to disrupt the interaction between PD1 and PDL1, such as the expression of (a) PD1 fusion to the CD28 costimulatory domain to convert the inhibitory signal into stimulation, (b) PD1 RNA interference, and (c) the expression of a secreted PD1 Fc fragment that binds to PD-L1 on tumor cells [54,55,56]. Administration of immune checkpoint inhibitors, neutralizing monoclonal antibodies against CTLA4 and PD1, has been shown to prevent the suppression of CAR T cells in many solid tumors [57].

## 8. CAR Clinical Trials in AML

**Figure 3:**
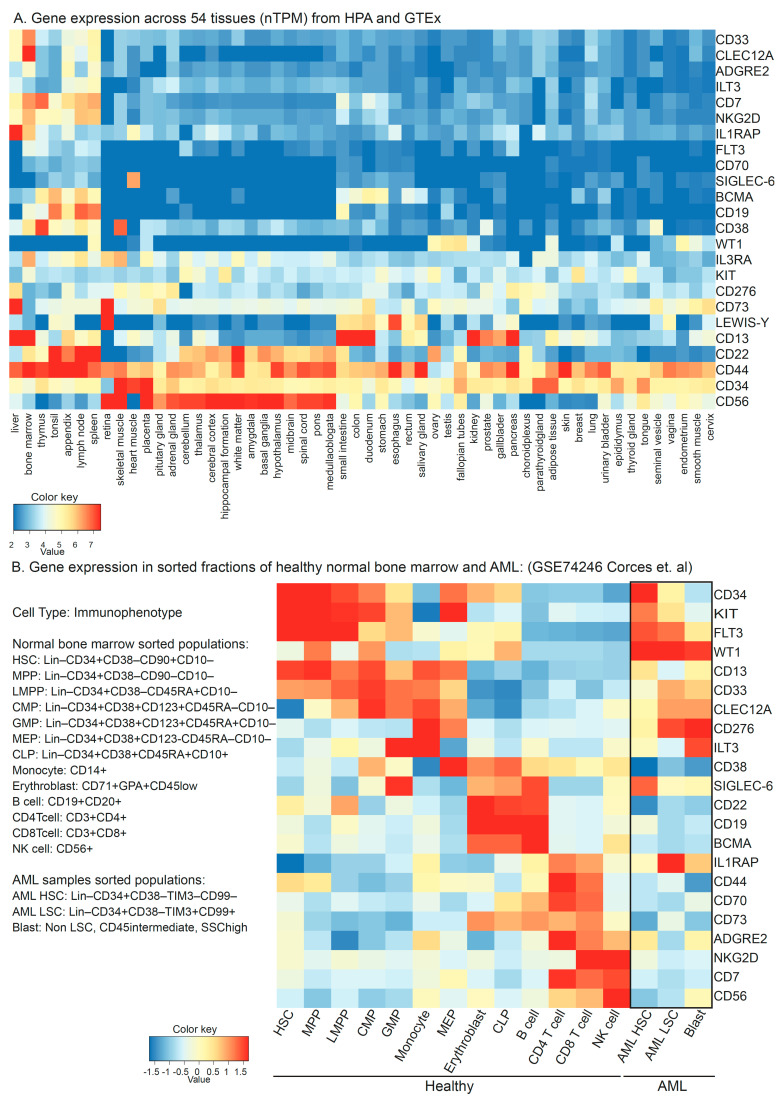
(**A**) Transcript-level expression of CAR targets in AML across 54 normal tissues in a consensus dataset retrieved at https://www.proteinatlas.org/ (accessed on 1 June 2023). (**B**) Expression of potential CAR targets at the mRNA level in sorted cell populations of normal healthy marrows from 12 donors and AML samples from 9 patients in single-cell RNA sequencing experiment submitted at gene expression omnibus GSE74246. HSC: Hematopoietic Stem Cells, MPP: Multipotent Progenitor, LMPP: Lymphoid–Myeloid Primed Progenitors, CMP: Common Myeloid Progenitors, GMP: Granulocyte–Monocyte Progenitors, MEP: Megakaryocyte–Erythrocyte Progenitors, and CLP: Common Lymphoid Progenitor.

An efficient and specific ‘on-target’ CAR-mediated tumor cell killing depends on the antigen against which the CAR is generated, the ability of the CAR cell to reach and recognize the malignant cells, and the microenvironment of the given tumor. Since AML is a bone marrow and blood disorder, low circulation of the CAR T cells is not likely to be a challenge, and CAR T efficacy is in large part limited to the features of the antigen. It is important that the antigen against which the CAR is generated (a) expresses abundantly and differentially on the surface of tumor cells compared to the normal cells of the same tissue, (b) shows minimal to no expression on the surface of normal cells across different tissues, and (c) does not express on the surface of T cells. So far, CAR T therapy has been most successful in the treatment of B cell malignancies, and the first recipient of CD19-targeting CAR T therapy was recently reported to be in complete remission (CR) for a decade [58]. The six US FDA-approved CAR products are for B cell malignancies, where four of those products target CD19 and two are against B Cell Maturation Antigen (BCMA) [47]. Both CD19 and BCMA are B cell differential antigens. B cells are specialized APCs that produce neutralizing antibodies. In the event of B cell depletion, immunoglobulins can be externally given, and the myeloid cells can work as bona fide APCs without causing generalized hemotoxicity. The availability of commercial CAR products, large amounts of real-world data, and clinical guidelines prove the efficacy of CAR T therapy in relapsed/refractory (R/R) B-ALL [59]. Unfortunately, we have not yet arrived at a CAR T in AML that matches this scenario, as a universal antigen for all AML subtypes and cells has not yet been identified. Given the disease heterogeneity and considering the danger of generalized myelotoxicity when targeting myeloid lineage cells, a more differential strategy may be required to make CAR therapy work in this disease. As of June 2023, 92 clinical trials are listed on the clinicaltrials.gov website that use CAR treatment in AML (also see Table S1). These trials include about 24 antigens, which can be divided into groups based on their expression patterns (overexpressed myeloid, repurposed or aberrant lymphoid, or neoantigens) and have been extensively reviewed in the literature [7,43,60,61,62,63]. The expression of these antigens at the transcript level in different tissues (Figure 3A; data from the human proteome atlas [64] and GTEx consortium [65], downloaded from proteinatlas.org and prepared using iDEP [66]) shows potential antigen distribution in normal cells. The expression of these antigens at mRNA levels in sorted lineage fractions from 12 healthy bone marrow and 9 AML samples [67] (Figure 3B) suggests only limited specificity for leukemic cells for WT1, but not for other antigens. We will briefly highlight some of the more prominent CAR target antigens used in AML:

**CD33**: CD33 is a transmembrane receptor, also known as sialic acid-binding immunoglobulin-related lectin (SIGLEC-3). CD33 is almost always expressed on AML blasts with 85–95% positivity and is an important diagnostic immunophenotypic marker for AML in combination with CD13 and myeloperoxidase. It is also considered an independent prognostic factor in AML [68]. Due to its abundant expression in AML, it has been considered an excellent target for therapeutic intervention using the blocking antibody gemtuzumab ozogamicin [69], bispecific T cell engagers (BiTE) [70], and CAR cells [71,72]. We identified 22/92 CAR trials targeting CD33 in AML, of which in five trials it is used in dual/multi-CAR in combination with either CD123, CLL1, or others, and in two it is one of several targets against which CAR is proposed. Although expression of CD33 is negligible outside the blood-forming tissues, it is expressed in almost all stages of myelopoiesis, and myelotoxicity has been a concern.

**CD123/IL3RA**: The IL3 receptor alpha chain is a transmembrane protein that is aberrantly over-expressed in AML and a few other hematological malignancies but has limited expression in cells of normal hematopoiesis [73]. It is expressed on the surface of the AML blast in about 77% of all the AML cases [74] and is the most common target of CAR in AML, as 25 of the 92 clinical trials have enlisted it. Three of these twenty-five trials incorporate a universal CAR system, and two trials use a combinatorial approach with other antigens.

**CLL1/CLEC12A**: C-type lectin-like molecule-1 is a transmembrane glycoprotein that expresses on the surface of AML blasts in about 77–92% of cases. CLL1 is not expressed on normal tissue of non-hematological origin, and within the normal hematopoietic system, it is absent from hematopoietic stem cells (HSC) but expresses along the myeloid differentiation trajectory and has a higher expression on leukemic stem cells (LSC) compared to HSC [75]. Its differential overexpression on LSC has rendered it a preferred combinatorial target in CAR therapy [76], as it is currently being used in eleven clinical trials, out of which five used it with other targets in combination to effectively target blast and LSC simultaneously. Apart from the CAR, CLL1 is also a therapeutic candidate for antibody drug conjugate (ADC) in AML [77,78].

**FLT3/CD135**: FLT3/CD135 is an Fms-like tyrosine kinase-3 is a class II tyrosine kinase receptor that is expressed on the surface of most hematopoietic stem and progenitor cells, where it plays a crucial role in signaling through Flt ligand (FL). High expression of FLT3 is detected on leukemic blasts in about 80–100% of AML cases. FLT3 is most studied for its recurring mutations in AML, where the mutations causing internal tandem duplication (ITD) in the juxtamembrane domain (JD) account for about 20% of AML and about 5–10% of cases harbor point mutations in the tyrosine kinase domain (TKD). ITD mutations are known to amplify FLT3 signaling through several downstream pathways and are a known prognostic marker in AML [79]. FLT3 is a key therapeutic target of several tyrosine kinase inhibitors (TKI), BiTE, and CAR strategies in AML [80,81]. The current ELN management guidelines suggest the usage of Midostaurin during induction, maintenance, and consolidation for AML patients with FLT3 mutations who are fit for intensive chemotherapy, and for patients unfit for an intensive regimen, gilternib as a single agent is recommended [8]. Currently, FLT3 is a candidate for six clinical trials with a lot of commercial interest, as four out of six trials use TAA05 from PersonGen or AMG553 from Amgen.

**NKG2D/CD314**: NKG2D/CD314 is a homodimeric receptor on NK and T cells that is encoded by the killer cell lectin-like receptor K1 (KLRK1) gene. NKG2D has a broad specificity for ligands such as MHC class I polypeptide-related sequences A and B (MICA and MICB) and six members of the UL16-binding glycoproteins 1–6 (ULBP1–6) that are expressed on the surface of AML blasts in about 67–100% of AML cases, which makes CAR cells expressing NKG2D a potent AML killer [82]. Engagement of NKG2D on the immune cell surface with NKG2D ligand on tumor cells leads to immune activation and tumor cell killing. Low expression of NKG2DL is a characteristic feature of LSC, and its downregulation is a major cause of relapse in AML [83]. Considering the strong potential of NKG2D CAR in AML, several approaches, such as AML treatment with DNA hypomethylating agents and small-molecule-based activation of KLF4, have been identified for improved immune surveillance through NKG2D CAR [84,85].

**CD38**: CD38, also known as ADP-Ribosyl Cyclase 1 or NAD (+) Nucleosidase, is a cell surface receptor and catalytic enzyme that is moderately expressed on myeloid and erythroid progenitors but predominantly expressed on the surface of B lymphocytes, with the highest expression on plasma cells. A moderate to variable expression of CD38 is seen on the surface of AML blasts, and LSCs are known to be CD38-negative, which poses a potential problem for this approach. The success of anti-CD38 antibody daratumumab in multiple myeloma (MM) and B malignancies corroborated preclinical studies in AML, which showed significant induction of apoptosis in AML cell lines and primary samples [86,87]. Due to their limited expression in normal hematopoietic cells, CARs against CD38 can be a potential solution to several hematologic neoplasms, including AML, with limited toxicity [88,89]. As of now, six clinical trials use CD38 CAR, where in two trials the CART38 product is being used and one trial proposes the use of a multi-CAR strategy. 

**CD19**: CD19 is a B cell surface receptor that is often aberrantly expressed on leukemic blasts in AML. Lymphoid cell surface markers such as CD7, CD56, and CD19 are frequently reported for aberrant expression on myeloid blasts in AML [90,91,92]. CD19 CARs have been approved by the FDA for their use in B cell malignancies. With their limited generalized toxicity and efficient target killing, CD19 CAR is a potential therapeutic option in CD19-positive AML. 

**CD7**: CD7 is a lymphoid-associated antigen that is restricted to T and NK cells and is an attractive target for intervention in T cell malignancies. Aberrant expression of CD7 on the surface of leukemic blasts in about 20% AML cases and its absence in normal myeloid cells make it a potential immunotherapy target in AML [93,94,95]. At the time of writing this review, CD7 was the CAR target candidate in five clinical trials in AML. Since CD7 is expressed abundantly on T and NK cells, in order to prevent CAR T cells killing each other, fratricide-resistant CAR strategies have been developed and are proposed in one clinical trial, NCT05377827, for AML.

**CD276/B7-H3**: CD276/B7-H3 is a transmembrane and soluble immune checkpoint molecule that is transcribed in a variety of cells, but its protein is reported to be expressed only in a very few normal cells, such as activated dendritic cells. A high level of B7 expression is observed on various cancer cells, including AML blasts, with about 60% positivity for CD276. High-level CD276 expression is correlated with poor outcomes in AML [96]. Inhibition of CD276 is reported to enhance AML cell killing by CAR T and NK cells [97,98]. As of now, three clinical trials incorporate the use of CAR against CD276 in relapsed refractory AML with CD276 positive blasts. 

Other than the antigens listed above, CD97, IL1RAP, Lewis Y, ILT3, SIGLEC-6, WT1, MUC1, and the splice variant of CD44 known as CD44v6 are considered myeloid-associated antigens suitable for CAR targeting in clinical trials. Two clinical trials, NCT03291444 and NCT03473457, propose CD34 and CD117 CAR, among several other antigens. Since CD34 and CD117 are expressed at high levels in HSCs and are crucial for normal hematopoiesis, careful consideration of these antigens in CAR strategy is suggested. CD70 and CD73 are lymphoid-repurposed antigens that are seen as potential CAR antigens in positive AML cases.

## 9. Improving CAR T Cell Therapy One Step at a Time

At first glance, a common theme of CAR therapy in AML is characterized by the variety of target antigens against which the receptor is developed. This may reflect the heterogeneity within the disease or a lack of prominent AML-specific antigens that can serve as targets. It appears that the field is still trying to provide an answer to this problem in multiple small trials rather than large-scale, multi-centric clinical trials. However, lessons can be learned from these studies. A meta-analysis that included 57 patients from 13 original reports (10 trials and 3 case reports) of CAR therapy for intervention in R/R AML reported complete remission (CR) in 38.5% (22 of 57, estimated pooled incidence 49.5%) patients. However, only one patient was reported to be free of cytogenetic disease for more than 20 months, and most of the patients either relapsed within six months or were subjected to further HCT if they remained negative for minimal residual disease (MRD) for up to four weeks after CAR infusion. Due to the huge variation in the endpoint set up in different studies and the small number of patients per trial, a cumulative overall survival could not be calculated. Pooled toxicity incidence surpassed the fraction of complete response as CRS was reported in 43.8% (25 of 57, pooled incidence of 54.4%) patients, and two deaths were reported due to grade IV Graft-versus-Host Disease (GvHD). The meta-analysis also included findings from an activated NK-92 clinical trial without CAR target NCT00900809, where disease progression was reported in all the patients [99]. This analysis revealed five major challenges across the included studies—(a) disease heterogeneity: different studies have different inclusion criteria, patients with different mutational burdens, and previous treatments; (b) lack of a universal target: although CD33, CLL1, CD123, and NKG2D were the main targets, most studies reported less than 10 patients treated with CAR targeted against one antigen, which makes it difficult to draw conclusions about the efficacy of a universal CAR product; (c) toxicity: due to the lack of a ‘safe target’, almost half of the patients developed CRS, five reported the development of immune effector cell-associated neurotoxicity syndrome (ICANS), and other toxicities were reported in individual studies; (d) limited off-the-shelf option: although allogeneic T cell products seem like a lucrative option, the meta-analysis observed that patients who developed severe GvHD were given allogeneic CAR T products; and (e) modest response due to CAR exhaustion: resulting in CAR therapy only being used as a bridge to HSCT rather than a curative intervention. 

Some additional lessons can also be drawn from recently completed trials. In March 2023, Celyad Oncology (Belgium) released results from their multi-center THINK study, with the infusion of an autologous CAR T product (CYAD-01) against NKG2D given to 16 R/R AML, MDS, and MM patients who had received at least one line of previous treatment [100]. Ligands for NKG2D are expressed on a variety of malignant cells and are generally absent from normal cells. As expected, the study reported no myelosuppression, no neurotoxicity, and limited cytokine release syndrome (CRS). However, two major challenges of this trial were (a) manufacturing failure in patients with high leukemic burden and (b) limited response due to the lack of CAR persistence. Prior lymphodepletion and bridging therapy to overcome the cytokine sink caused by the high number of blasts and optimization of the CAR protocol to select functional T cells instead of memory cells have been suggested to overcome these challenges [101]. Some of these challenges are currently addressed with the next-generation CAR design, as outlined above, and the use of off-the shelf options, but that alone may not be sufficient.

While there is nothing that can be done to overcome the challenge of disease heterogeneity and thus antigen diversity, many efforts are ongoing to find a ‘safe target’ in AML. In a notable study that a used single-cell transcriptomics atlas from 15 AML patients and 9 healthy individuals, the authors identified Colony Stimulating Factor 1 Receptor (CSF1R) and CD86 as potential safe and effective targets for CAR therapy in AML. The two antigens are preferentially expressed on the surface of AML cells and lack expression on normal cells of other tissues and normal hematopoiesis [102]. During the 2022 meeting of the American Society of Hematology, two different groups reported the pre-clinical development of CD84 targeting CAR in pediatric and adult AML [103,104]. In a non-conventional approach, a preclinical study reported the use of a TCR mimic against the peptide–HLA complex to target Preferentially Expressed Antigen in Melanoma (PRAME), which is expressed by several AML cell lines, primary AML, and other malignancies but has restricted expression in cells of normal hematopoiesis and is referred to as cancer testis antigen [105]. A recent review outlines several other biomarkers that are in preclinical studies for evaluation of their safety and efficacy in AML [106]. It seems apparent that the search for the ideal CAR target(s) in AML is still ongoing and may limit its immediate success in this disease. 

CAR T exhaustion is another major challenge in AML (and other cancers) that is pertained to the T cell intrinsic pathways and extrinsic features such as tumor microenvironment and loss of signaling due to antigen depletion. PD1 blockade, NR4A depletion, TGF-β signaling blockade, CRISPR-mediated deletion of CBLB to overcome cell intrinsic pathways, and changes in CAR design such as self-driving CARs with activation signaling motifs and switchable or split CAR designs are suggested to overcome some of these challenges [56]. Pretreatment of CAR with cytokines in addition to other immune effector cells can also help overcome the limited immune response. In one trial (NCT03291444), it is proposed to use intradermal injection of dendritic cells specific for epidermal growth factor receptor kinase substrate (EPS8) or Wilms tumor 1 (WT1) after CAR T infusion. Eleven clinical trials are using a combinatorial CAR strategy to overcome the challenge of antigen-depleted relapse, where CLL1 is targeted along with CD33, CD123, or both. Inspired by the initial success of the multi-CAR T trial in lymphomas [107], the investigators have proposed using a similar strategy for multi-CAR T in AML (trial ID: NCT03222674).

To address the challenge of CAR-related toxicities, at least two clinical trials propose a suicide switch in their CAR products. A CAR design by AGC Biologics (Italy) uses the HSV-TK Mut2 gene, which can be selectively activated in cases of severe toxicity through the administration of ganciclovir (trial ID: NCT04097301), to shut off the CD44v6-directed CAR T. 2seventy bio (Cambridge, MA, USA) has developed a system with Dimerizing Agent Regulated Immunoreceptor Complex (DARIC) (trial ID: NCT05105152) where rapamycin can be used to induce dimerization of FRB-FKBP12 to inhibit mTOR signaling and induce cell death in CAR T directed against CD33. Other preclinical strategies to overcome the CAR-mediated toxicities through the incorporation of ON and OFF switches in CAR products for their application in AML have been recently reviewed by Atilla and Benabdellah [108].

NK cell-based CARs are suggested to have several advantages over T cell-based CARs. Currently, there are 10 clinical trials using CAR NK, where 2 trials propose using the off-the-shelf NK-92 cell line, another 2 uses off-the-shelf donor-derived allogeneic NK, and 1 uses cord-blood-derived NK cells for CAR manufacturing. The advantages of the CAR NK approach are limited toxicity due to a shorter life span in the circulation, minimal GvHD, no functional exhaustion, and CAR-independent mechanisms through the activation of immune effectors for tumor cell killing, in addition to the off-the-shelf manufacturing options [109]. Nkarta (San Francisco, CA, USA) recently released data (nkartatx) from their clinical trial (NCT04623944) that used NKG2D-based CAR, which is an off-the-shelf, allogeneic donor derived NK cell product with membrane-bound IL-15 (NKX101). Data from 36 R/R AML patients in total were reported, where lymphodepletion prior to CAR infusion was carried out using a combination of either fludarabine–cyclophosphamide (Flu/Cy) in 30 patients or fludarabine–cytarabine (Flu/Ara-C) in 6 patients. The Flu/Ara-C combination is known to induce expression of NKG2D ligands in cancer cells. In the Flu/Cy cohort, responses were observed only with the highest dose, and complete response with hematological recovery was reported in 17% of patients. Complete remission with incomplete count recovery (CRi) was reported in 4 out of 18 patients. Among toxicities, 12% reported CRS, and only one case of grade ≤ 2 ICANS and no cases of GvHD were reported in this cohort. In the R/R AML patient cohort that received Flu/Ara-C prior to NKX101, 3 of 6 achieved complete response with hematological recovery, and CRi was reported in 67% (4 out of 6). None of the CAR-related toxicities were observed. The most common higher grade adverse events in all patients were myelosuppression and infection. The lower frequency of CAR-related toxicities and the absence of GvHD makes an NK-based CAR significantly safer than a T-based CAR. 

## 10. Conclusions

Although there are several challenges that remain to be addressed before CAR therapy can be used as a singular intervention in AML, the potential of CAR treatment, especially for R/R AML cases with poor prognostic mutations, complex karyotypes, and higher disease burden, is undeniable. Alternatively, CAR therapy could also be established as bridge therapy before HSCT. Due to the paucity of AML-exclusive targets, myeloablation remains a major concern for CAR therapy in AML. This is true in particular for CAR targeting CD33 and CD123, both of which are broadly expressed in the myeloid compartment. Strategies to control CAR therapy through inducible receptor activity would be helpful here and are likely to be required in AML. Another interesting strategy are universal CAR systems that would allow for the use of multiple receptors to adapt to the patients’ unique antigen profiles. Receptors could be used in combination to delay or avoid resistance. As pointed out above, there are also some differences between T cells and NK cells, but the jury is still out on whether either system is truly more advantageous to carry the CAR treatment. Finally, the role of the immune microenvironment is complex, and there is potential to improve CAR therapy with appropriate cytokines or additional receptors. Despite the disappointing shortcomings of CAR therapy in AML, initial studies clearly demonstrate at least some measurable activity in a considerable number of patients, and in the near future, with further innovations in this field, it might be offered as an intervention strategy. 

## Data Availability

Clinical trial data were downloaded from clinicaltrials.gov. The AML survival rate was taken from SEER*Explorer: an interactive website for SEER cancer statistics (Internet). Surveillance Research Program, National Cancer Institute; 19 April 2023. (Updated: 8 June 2023; cited 27 June 2023.) Available from https://seer.cancer.gov/statistics-network/explorer/ (accessed on 1 June 2023). Data source(s): SEER Incidence Data, November 2022 Submission (1975–2020), SEER 22 registries (excluding Illinois and Massachusetts). Expected Survival Life Tables by Socio-Economic Standards. Consensus transcriptomics data were downloaded from proteinatlas.org. Single-cell transcriptomics data were taken from GSE74246.

## Supplementary Materials

The following supporting information can be downloaded at: https://www.mdpi.com/article/10.3390/hematolrep15040063/s1, Table S1: Clinical trials using CAR in AML identification numbers from clinicaltrials.gov (accessed on 1 June 2023).
