# Supplementary material for: Chimeric Antigen Receptor T Cell Therapy in Acute Myeloid Leukemia: Trials and Tribulations
Source: Hematol Rep. 2023 Nov 12;15(4):608–26. doi: 10.3390/hematolrep15040063 (PMC10660693; doi:10.3390/hematolrep15040063)
Supplement: Supplementary file 1 [file hematolrep-15-00063-s001.zip › hematolrep-2537644-supplementary.pptx]

## Slide 1
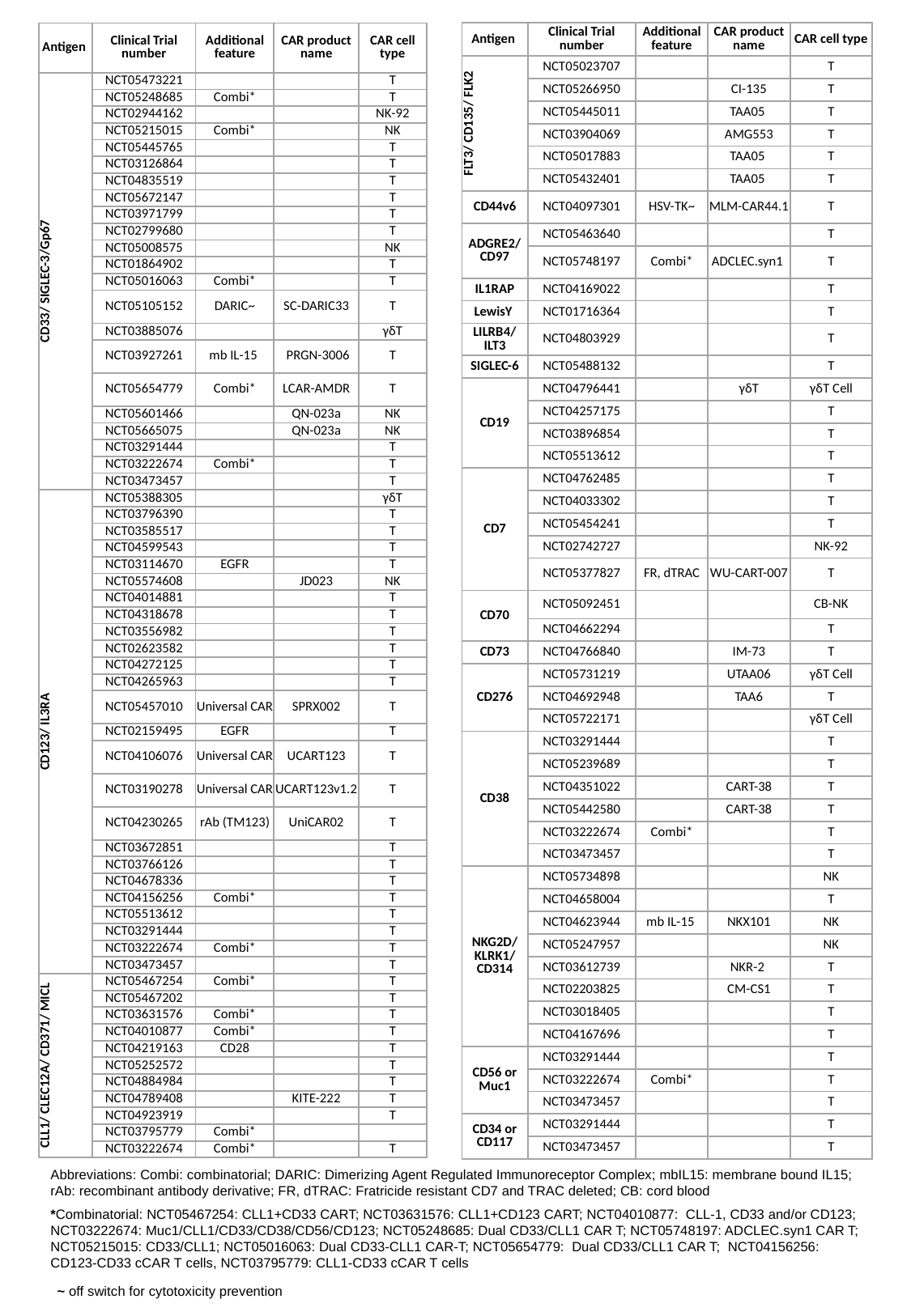

| Antigen | Clinical Trial number | Additional feature | CAR product name | CAR cell type |
| --- | --- | --- | --- | --- |
| FLT3/ CD135/ FLK2 | NCT05023707 | | | T |
| | NCT05266950 | | CI-135 | T |
| | NCT05445011 | | TAA05 | T |
| | NCT03904069 | | AMG553 | T |
| | NCT05017883 | | TAA05 | T |
| | NCT05432401 | | TAA05 | T |
| CD44v6 | NCT04097301 | HSV-TK~ | MLM-CAR44.1 | T |
| ADGRE2/ CD97 | NCT05463640 | | | T |
| CD97 | NCT05748197 | Combi\* | ADCLEC.syn1 | T |
| IL1RAP | NCT04169022 | | | T |
| LewisY | NCT01716364 | | | T |
| LILRB4/ ILT3 | NCT04803929 | | | T |
| SIGLEC-6 | NCT05488132 | | | T |
| CD19 | NCT04796441 | | γδT | γδT Cell |
| | NCT04257175 | | | T |
| | NCT03896854 | | | T |
| | NCT05513612 | | | T |
| CD7 | NCT04762485 | | | T |
| | NCT04033302 | | | T |
| | NCT05454241 | | | T |
| | NCT02742727 | | | NK-92 |
| | NCT05377827 | FR, dTRAC | WU-CART-007 | T |
| CD70 | NCT05092451 | | | CB-NK |
| | NCT04662294 | | | T |
| CD73 | NCT04766840 | | IM-73 | T |
| CD276 | NCT05731219 | | UTAA06 | γδT Cell |
| | NCT04692948 | | TAA6 | T |
| | NCT05722171 | | | γδT Cell |
| CD38 | NCT03291444 | | | T |
| | NCT05239689 | | | T |
| | NCT04351022 | | CART-38 | T |
| | NCT05442580 | | CART-38 | T |
| | NCT03222674 | Combi\* | | T |
| | NCT03473457 | | | T |
| NKG2D/ KLRK1/ CD314 | NCT05734898 | | | NK |
| | NCT04658004 | | | T |
| | NCT04623944 | mb IL-15 | NKX101 | NK |
| | NCT05247957 | | | NK |
| | NCT03612739 | | NKR-2 | T |
| | NCT02203825 | | CM-CS1 | T |
| | NCT03018405 | | | T |
| | NCT04167696 | | | T |
| CD56 or Muc1 | NCT03291444 | | | T |
| | NCT03222674 | Combi\* | | T |
| | NCT03473457 | | | T |
| CD34 or CD117 | NCT03291444 | | | T |
| | NCT03473457 | | | T |
| Antigen | Clinical Trial number | Additional feature | CAR product name | CAR cell type |
| --- | --- | --- | --- | --- |
| CD33/ SIGLEC-3/Gp67 | NCT05473221 | | | T |
| | NCT05248685 | Combi\* | | T |
| | NCT02944162 | | | NK-92 |
| | NCT05215015 | Combi\* | | NK |
| | NCT05445765 | | | T |
| | NCT03126864 | | | T |
| | NCT04835519 | | | T |
| | NCT05672147 | | | T |
| | NCT03971799 | | | T |
| | NCT02799680 | | | T |
| | NCT05008575 | | | NK |
| | NCT01864902 | | | T |
| | NCT05016063 | Combi\* | | T |
| | NCT05105152 | DARIC~ | SC-DARIC33 | T |
| | NCT03885076 | | | γδT |
| | NCT03927261 | mb IL-15 | PRGN-3006 | T |
| | NCT05654779 | Combi\* | LCAR-AMDR | T |
| | NCT05601466 | | QN-023a | NK |
| | NCT05665075 | | QN-023a | NK |
| | NCT03291444 | | | T |
| | NCT03222674 | Combi\* | | T |
| | NCT03473457 | | | T |
| CD123/ IL3RA | NCT05388305 | | | γδT |
| | NCT03796390 | | | T |
| | NCT03585517 | | | T |
| | NCT04599543 | | | T |
| | NCT03114670 | EGFR | | T |
| | NCT05574608 | | JD023 | NK |
| | NCT04014881 | | | T |
| | NCT04318678 | | | T |
| | NCT03556982 | | | T |
| | NCT02623582 | | | T |
| | NCT04272125 | | | T |
| | NCT04265963 | | | T |
| | NCT05457010 | Universal CAR | SPRX002 | T |
| | NCT02159495 | EGFR | | T |
| | NCT04106076 | Universal CAR | UCART123 | T |
| | NCT03190278 | Universal CAR | UCART123v1.2 | T |
| | NCT04230265 | rAb (TM123) | UniCAR02 | T |
| | NCT03672851 | | | T |
| | NCT03766126 | | | T |
| | NCT04678336 | | | T |
| | NCT04156256 | Combi\* | | T |
| | NCT05513612 | | | T |
| | NCT03291444 | | | T |
| | NCT03222674 | Combi\* | | T |
| | NCT03473457 | | | T |
| CLL1/ CLEC12A/ CD371/ MICL | NCT05467254 | Combi\* | | T |
| | NCT05467202 | | | T |
| | NCT03631576 | Combi\* | | T |
| | NCT04010877 | Combi\* | | T |
| | NCT04219163 | CD28 | | T |
| | NCT05252572 | | | T |
| | NCT04884984 | | | T |
| | NCT04789408 | | KITE-222 | T |
| | NCT04923919 | | | T |
| | NCT03795779 | Combi\* | | |
| | NCT03222674 | Combi\* | | T |
Abbreviations: Combi: combinatorial; DARIC: Dimerizing Agent Regulated Immunoreceptor Complex; mbIL15: membrane bound IL15; rAb: recombinant antibody derivative; FR, dTRAC: Fratricide resistant CD7 and TRAC deleted; CB: cord blood
*Combinatorial: NCT05467254: CLL1+CD33 CART; NCT03631576: CLL1+CD123 CART; NCT04010877: CLL-1, CD33 and/or CD123; NCT03222674: Muc1/CLL1/CD33/CD38/CD56/CD123; NCT05248685: Dual CD33/CLL1 CAR T; NCT05748197: ADCLEC.syn1 CAR T; NCT05215015: CD33/CLL1; NCT05016063: Dual CD33-CLL1 CAR-T; NCT05654779: Dual CD33/CLL1 CAR T; NCT04156256: CD123-CD33 cCAR T cells, NCT03795779: CLL1-CD33 cCAR T cells
~ off switch for cytotoxicity prevention
